# In Situ Electron Microscopy of Lactomicroselenium Particles in Probiotic Bacteria

**DOI:** 10.3390/ijms17071047

**Published:** 2016-06-30

**Authors:** Gabor Nagy, Gyula Pinczes, Gabor Pinter, Istvan Pocsi, Jozsef Prokisch, Gaspar Banfalvi

**Affiliations:** 1 Department of Biotechnology and Microbiology, University of Debrecen, Debrecen 4010, Hungary; bigdegu@gmail.com (G.N.); pigyu@freemail.hu (G.P.); pintyo@gmail.com (G.P.); ipocsi@gmail.com (I.P.); 2 Department of Animal Breeding, University of Debrecen, Debrecen 4010, Hungary; jprokisch@agr.unideb.hu

**Keywords:** transmission electronmicroscopy, lactomicroSel, nanoparticles, lactobacilli, *Streptococcus thermophilus* (*S. thermophilus*)

## Abstract

Electron microscopy was used to test whether or not (a) in statu nascendi synthesized, and in situ measured, nanoparticle size does not differ significantly from the size of nanoparticles after their purification; and (b) the generation of selenium is detrimental to the bacterial strains that produce them. Elemental nano-sized selenium produced by probiotic latic acid bacteria was used as a lactomicroselenium (lactomicroSel) inhibitor of cell growth in the presence of lactomicroSel, and was followed by time-lapse microscopy. The size of lactomicroSel produced by probiotic bacteria was measured in situ and after isolation and purification. For these measurements the TESLA BS 540 transmission electron microscope was converted from analog (aTEM) to digital processing (dTEM), and further to remote-access internet electron microscopy (iTEM). *Lactobacillus acidophilus* produced fewer, but larger, lactomicroSel nanoparticles (200–350 nm) than *Lactobacillus casei* (*L. casei*), which generated many, smaller lactomicroSel particles (85–200 nm) and grains as a cloudy, less electrodense material. *Streptococcus thermophilus* cells generated selenoparticles (60–280 nm) in a suicidic manner. The size determined in situ in lactic acid bacteria was significantly lower than those measured by scanning electron microscopy after the isolation of lactomicroSel particles obtained from lactobacilli (100–500 nm), but higher relative to those isolated from *Streptococcus thermopilus* (50–100 nm). These differences indicate that smaller lactomicroSel particles could be more toxic to the producing bacteria themselves and discrepancies in size could have implications with respect to the applications of selenium nanoparticles as prebiotics.

## 1. Introduction

Fermentation technology has been adapted to use a mixture of *Lactobacillus* species and *Streptococcus thermophilus* (*S. thermophilus*) to produce probiotic lactomicroselenium (lactomicroSel) particles under laboratory conditions [1,2]. Earlier subacute toxicity studies in mice performed by the per os administration of 0.5 and 50 ppm selenate, selenite, SelPlex, nanoSel, and lactomicroSel selenium sources revealed that lactomicroSel was the only Se species that caused neither traceable accumulation in different organs nor nephrotoxicity. Only lactomicroSel was recommended for selenium supplementation [3,4]. In addition to the in vivo toxicology studies, the in vitro toxicology of lactomicroSel in human skin keratinocytes (HaCaT cells) between 0.5 and 5 ppm (0.5 and 5 mg/kg) did not exert inhibition on cell growth [4].

To visualize and measure the size of probiotic selenium particles our analog transmission electron microscope (TEM) has been converted to digital mode and its magnification was raised from 180,000× to 250,000× due to the application of digital photography with a charge-coupled device (CCD). High-resolution TEM proved to be advantageous to observe structural details and measure nanoparticles in situ inside the cells [5].

The aim of this study was (a) to determine the lowest inhibitory concentration of lactomicroSel, that causes measurable inhibition of cell growth; (b) to apply electron microscopy to estimate the size of lactomicroSel particles inside the cells and outside after isolation and purification by scanning electron microscopy; and (c) to find a relationship between nanoparticle size and cellular toxicity.

## 2. Results

### 2.1. Inhibition of Cell Growth by Lactomicroselenium (LactomicroSel)

The cellular toxicity of different selenium species including selenate, selenite, SelPlex, and nanoSel between 0.5 and 5 ppm was contrasted by the lack of growth inhibition of lactomicroSel in HaCaT cell cultures [4].

**Figure 1:**
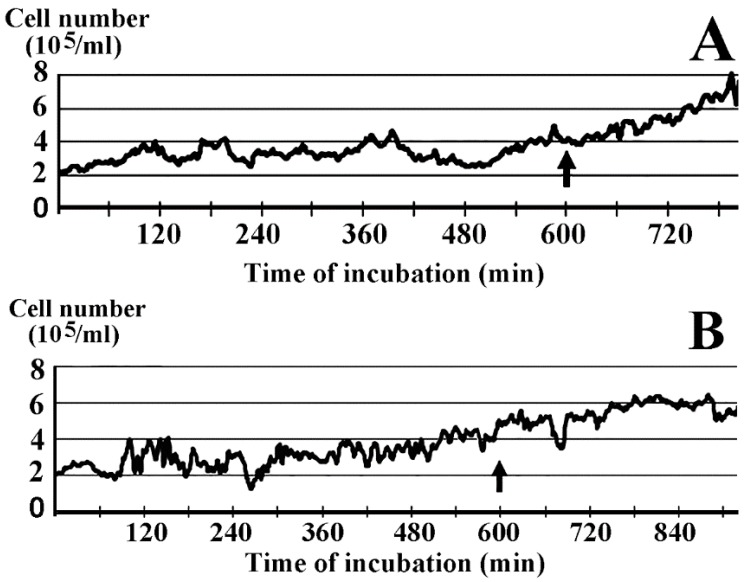
HaCaT cell growth curves in the presence and absence of lactomicroselenium (lactomicroSel) based on data of time-lapse image analysis. Two 5 mL cell cultures in 25 mL T-flasks were started by plating HaCaT cells at 2 × 10^5^ cells/mL. (**A**) Control cells were grown for 600 min, then 0.1 mL saline was added (arrow) and the growth continued up to 780 min; and (**B**) initial cell growth in the absence of selenium lasted for 600 min, then interrupted by the addition lactomicroSel (arrow) resulting in a 10 ppm lactomicroSel final concentration and the growth continued for up to 920 min.

In vitro and in vivo experiments led to the conclusion that the toxicity order of selenium species was: selenate > selenite > SelPlex = nanoSel > lactomicroSel. In the presence of 1 ppm (1 mg/kg ≈ 1 mg/L ≈ 12.66 μM) inorganic nanoSel growth was inhibited, whereas probiotic lactomicroSel did not exert inhibition on HaCaT cell growth up to 5 ppm [4]. Figure 1 shows that the smallest lactomicroSel concentration that exerted measurable inhibition on cell growth was 10 ppm (~127 μM). Relative to the control (Figure 1A) cellular damage at 10 ppm lactomicroSel was reflected by the slower growth rate peaking at around 800 min (Figure 1B), when the control growth was still in full swing.

### 2.2. Scanning Electronmicroscopy of LactomicroSel Particles

**Figure 2:**
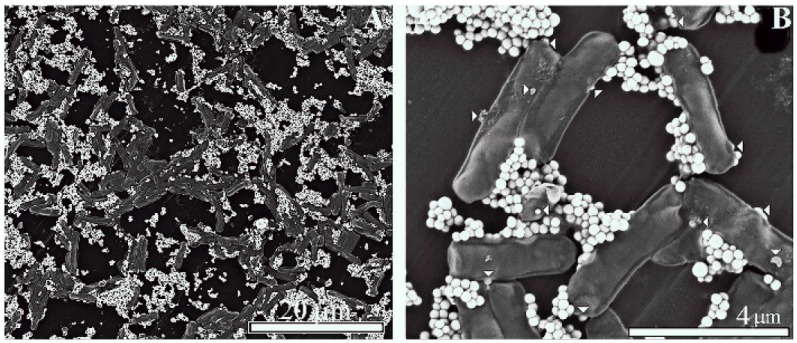
Scanning electron microscopy of lactomicroSel particles produced by *Lactobacillus casei.* (**A**) Visualization of lactomicroSel particles at low magnification. Scale bar, 20 µm; and (**B**) lactomicroSel particles at higher magnification. White arrows show the sites of bacilli where the nanoparticles are being excreted. Scale bar, 4 µm.

Scanning electron microscopy revealed that in order to obtain proper sized lactomicroSel particles was to select among those bacteria that are already being used in the food industry. Our new lactomicroSel product in this context meets the strict quality requirements regarding food supplements and additives. The liver-protective effect of *Lactobacillus acidophilus* (*L. acidophilus*) and *Lactobacillus casei* (*L. casei*), isolated from fresh cow milk [6], suggested that these two lactobacilli could be good candidates to produce probiotic lactomicroSel. That the technology using lactobacilli was more effective than the chemical synthesis is demonstrated by production of lactmicroSel in *Lactobacillus casei* resulting in a relatively regular and uniform size, and high purity of selenium spheres (150–400 nm, average ~250 nm in diameter) (Figure 2). The production was economical and faster, and parameters could be controlled. In the future we plan to use these novel lactomicroSel species as food supplements, forage additives, or plant nutritions [2], and it was important to answer another relevant question, namely, whether or not the size of lactomicroSel particles inside bacteria is similar to the size of these particles after their manipulation by isolation and purification. For the measurement of lactomicroSel particles inside the lactobacilli, transmission electron microscopy (TEM) was used.

### 2.3. Transmission Electron Microscopy

**Figure 3:**
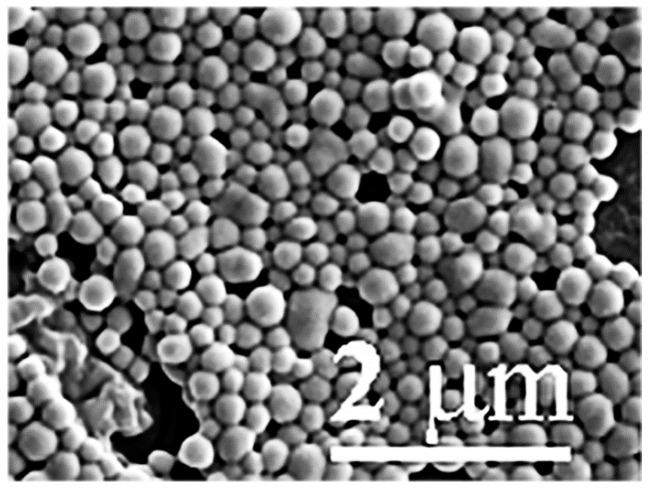
Size of lactomicroselenium particles measured by scanning electron microscopy after isolation and purification.

The diameter of selenium species referred to as lactomicroSel produced from yoghurt and sodium selenite by probiotic bacteria (*L. acidophilus*, *L. casei*, and *S. thermophilus*) after isolation and purification estimated by Scanning Electron Microscopy (SEM) was somewhat higher (100–500 nm) (Figure 3) than the size of the nanoparticles produced by *L. casei* alone (150–400 nm). This difference could come from the manipulation during isolation and purification or from the different particle size produced by different probiotic bacteria. To determine the size of nanomicroSe particles inside different probiotic bacteria we have used transmission electron microscopy. These TEM measurements in bacteria provided information regarding size and number of the lactomicroSel nanopartilces measured in lactobacilli in situ. The suicide production of thermophilomicroSe particles by *S. thermophilus* resulted in necrotic cell disruption.

**Figure 4:**
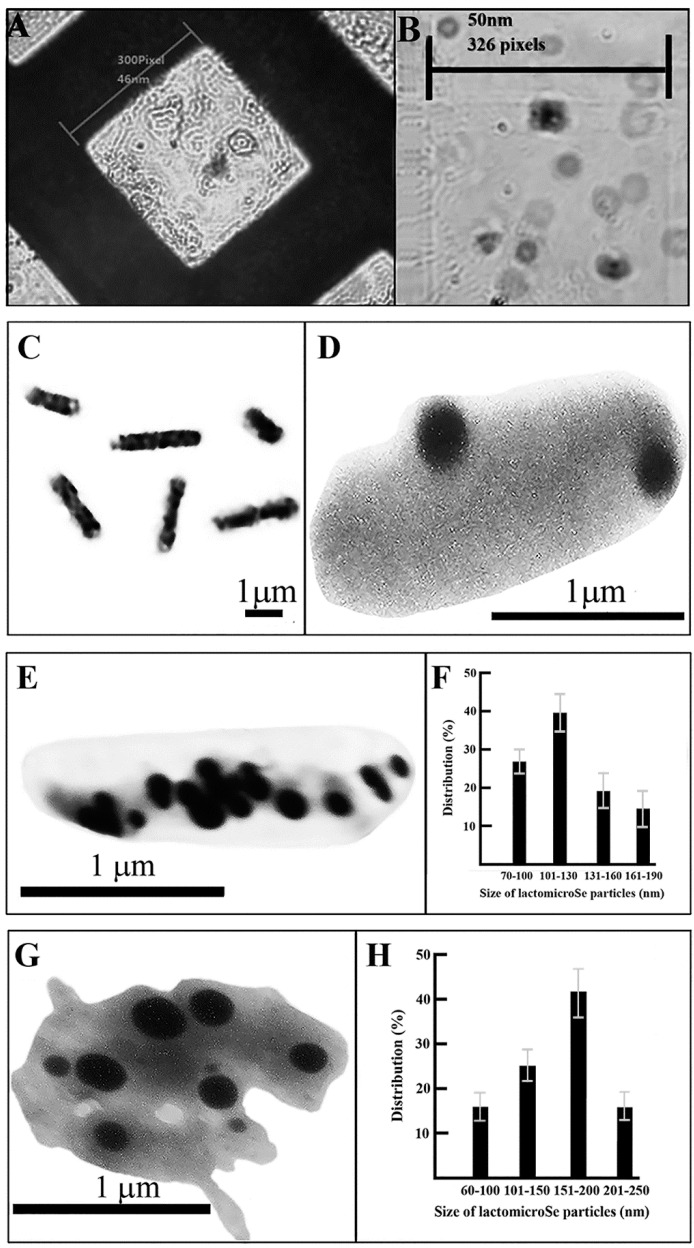
Transmission electron microscopy of lactomicroSel particles. Fermentation by probiotic yogurt bacteria (*L. acidophilus*, *L. casei*, and *S. thermophilus*) in the presence of sodium selenite was as decribed [2]. The lactomicroSel-containing bacilli were subjected to internet electron microscopy (iTEM). (**A**) Calibration of the transmission electron microscope. Individual squares of the TEM grid with an edge of about 300 pixels wide corresponded to 46 nm; (**B**) the edge of the small square in the Bürker chamber used for the measurement was 50 nm (326 pixels, 1 nm = 6.52 pixels), determined by the ImageJ software program; (**C**) light microscopy of *L. acidophilus* after fermentation and Gram staining; (**D**) lactomicroSel in a *L. acidophilus*; (**E**) iTEM of *L. casei* containing lactomicroSel nanoparticles; (**F**) size distribution of lactomicroSel particles in *L. casei*; (**G**) iTEM of disrupted *S. thermophilus* containing lactomicroSel particles; and (**H**) size distribution of lactomicroSel particles in *S. thermophiles.*

Before measuring the size of lactomicroSel particles, grid calibration was performed. This involved the following items: counting chamber, microscope slide with a red blood cell (average diameter 10 nm) sample, transmission electron microscope (TEM), and TEM sample grid (Figure 4A). The length of the edges of the smaller squares in the counting chamber were determined by the ImageJ software program (Figure 4B).

We have determined the size of probiotic bacteria used for yogurt production, namely *L. acidophilus* (0.5–1.1 × 1.2–2.5 µm), *L. casei* (0.6–1.1 × 1.5–4 µm), and *S. thermophilus* (0.6–1.2 µm) after the calibration of transmission electron microscope. The average size of the microbes *L. acidophilus*, *L. casei*, and *S. thermophilus* was 0.9 × 1.8, 0.6 × 2.6 and 0.82 µm, respectively. Comparable measurements performed earlier have shown that *L. acidophilus* was about 0.6–0.9 × 1.5 µm in size according to Bergey's manual. *L. casei* showed a cell size range between 0.7–1.1 × 2.0–4.0 µm [7]. The size of *S. thermophiles*, determined by scanning electron microscope, was between 1.07 and 1.21 µm [8].

After these measurements we have determined the size of lactomicroSel nanoparticles produced by these probiotic bacteria in the presence of sodium selenate. These particles could not be seen in *L. acidophilus* under light microscope (Figure 4C). Spherical nanoparticles produced during the fermentation of probiotic bacteria from sodium selenite were seen in *L. acidophilus* as few but larger lactomicroSel particles (210–350 nm) (Figure 4D). The thinner and longer *L. casei* cells contained more and smaller lactomicroSel particles (Figure 4E), with a size distribution between 85 and 200 nm (Figure 4F). It did not escape our attention that lactomicroSel was also present in *L. casei* cells as a less-dense cloudy substance (Figure 4E). The suicidic lactomicroSel production by a *S. thermophilus* coccus is demonstrated by its enlargement and disruption (Figure 4F). The size of lactomicroSel particles in *S. thermophilus* was between 70 and 250 nm (Figure 4G).

## 3. Discussion

TEM measurements of selenocompounds inside bacteria revealed that *L. acidophilus* produces fewer, but larger, lactomicroSel nanoparticles than *L. casei*, which generates many, small lactomicroSel particles and grains as a cloudy, less electrodense substance. *S. thermophilus* cells produce and accumulate selenoparticles that are toxic to the host cell, raising the question of bacterial cell death. The size of probiotic lactomicroSel particles inside the cells (60–350 nm) was smaller than those particles (100–500 nm) measured by SEM after isolation and purification, indicating that the manipulation of nanomicroSe may cause some aggregation. As smaller particles are likely to be more toxic, the moderate increase in size after the manipulation of microSe particles has no impact on their Se supplementation.

Suicide cell death was observed in lactomicroSel producing *S. thermophilus* bacteria. Programmed cell death could play an important role in the developmental processes of bacteria, e.g., lysis of *Bacillus subtilis* during sporulation [9,10], in fruiting body formation of vegetative cells of *Myxococcus xanthus* [9], ion transport leading to bacterial death in *S. pneumoniae* [11], and sequence specific mRNA cleavage in *E. coli* [10]. The concept of programmed cell death in bacteria has been met with criticism, but can no longer be ignored [12]. In agreement with these ideas bacterial cell death may also occur under toxic conditions. Due to the resistance and rigidity of the bacterial cell wall apoptotic shrinkage is less likely to be observed, but in *S. thermophilus* the toxicity of selenoparticles became visible and resulted in the disruption of bacteria.

## 4. Materials and Methods

### 4.1. Exposure of Cell Culture to LactomicroSel

The HaCaT cell line derived from human skin keratinocytes spontaneously transformed in vitro during long time incubation [13,14] was used to measure cytotoxicity. Cells were grown at 37 °C in RPMI-1640 medium (Sigma-Aldrich Kft., Budapest, Hungary) containing 10% fetal bovine serum (Hyclon, Logan, UT, USA). Cell cultures were started at an initial cell concentration of 2 × 10^5^/mL and grown for 10 h in the absence, and then in the presence, of lactomicroSel for 5 h.

### 4.2. Production of LactomicroSel

Studies during the production of yoghurt in our laboratory led to the discovery that certain bacteria species were able to transform the selenite at toxic concentrations into nano-sized, elemental selenium spheres via a so-far-unknown biochemical pathway. This was the first technology in which lactic acid and other probiotic bacteria produced elemental selenium in laboratory environment [2]. Lactomicroselenium was produced by probiotic bacteria during yoghurt fermentation with sodium nitrite by Bionanoferm Ltd., Debrecen, Hungary [1]. Yoghurt is normally produced by two lactic acid bacteria, namely *L. bulgaricus* and *S. thermophilus*. Some probiotic yoghurts contain *L. acidophilus, Bifidobacterium* spp., or other probiotic species. For the production of lactomicroSel solid sodium nitrite and, as inoculum, a mixture of three lactic acid bacteria strains, *L. acidophilus* (NCAIM B02085), *L. casei* (NCAIM B1147), and *S. thermophilus* (CNCM I-1670) obtained from the National Collection of Agricultural and Industrial Microorganisms, (Budapest, Hungary) isolated from Danon yoghurt (Danon, Alletown, PA, USA) were used. Briefly, the production of lactomicroSel took place in a rotary shaker at 37 °C for 48 h. At the end of the fermentation process, a Se-rich pink or red-colored yogurt was obtained. The yogurt was centrifuged for 5 min at 2000× *g* to get rid of most of the water. After decantation, the solid phase was placed into a 50–60 °C dryer for 16 h. Grinding was followed by mixing red lactomicroselenium into the feed. The final concentration of lactomicroSel was ~5 ppm with >95% of Se in the form of nanoparticles and <5% as organic Se.

### 4.3. Determination of Lactomicroselenium Concentration

Flame emission atomic absorption spectrometer (Thermo ICE 3000) and atomic fluorescencespectrometer (PSA Thermo-Fisher Excalibur, Waltham, MA, USA) served to determine the final selenium concentration of the prepared lactomicroSel samples. During the process samples were digested in the presence of nitric acid, hydrogen peroxide, and heated at 120 °C for 60 min. Digested samples were filtered and adjusted to 50 mL with distilled water. In the purified selenium samples the selenium concentration was between 200 and 500 mg/L, after lyofilisation the concentration of lactomicroSel batches varied between 2–3 g/kg, with >95% of Se in the form of nanoparticles and <5% as organic Se. For the visualization of lactomicroSel particles we used scanning and transmission electron microscopy. The size of the lactomicroSel was determined by a particle size analyzer (Malvern, Mastersizer 2000). The measurements were done in the laboratory of the Department of Solid State Physics, University of Debrecen, Debrecen, Hungary.

### 4.4. Transmission Electron Microscopy (TEM)

To operate the remote access internet electron microscopy (iTEM), our TESLA BS 540 TEM (Brno, Czechoslovakia) was equipped with a CCD camera by attaching it to the screen of the electron microscope. The transmission electron microscopy (TEM) diffraction patterns of microbes were measured at room temperature (*T*_0_) with TESLA BS 540 microscope operating at 80 kV with λ = 0.00418 nm. A computer-controlled electric power steering stage control unit allowed the precise identification of specimens on the stage plate. The digital conversion of the TESLA BS 540 transmission electron microscope involved:

(a) electric control rather than mechanical stage movement;
(b) application of digitazing device; and
(c) custom-built camera with Sony Exview-HAD CCD sensor (Sony Semiconductor Solutions Corporation, Tokyo, Japan).

Image processing was done by the National Health Institute ImageJ software bundle program, using custom-developed plugins and macros (available at: http://rsbweb.nih.gov/ij/docs/install/windows.html). Image sequences were first deflickered using a sequence stack histogram to avoid transient brightness changes between separate frames. After glutaraldehyde fixation lactomicroSel-containing bacilli were subjected to size measurement.

Internet transmission electronmicroscopy (iTEM) diffraction patterns from lactobacilli and *S. thermophilus* were measured at room temperature (*T*_0_) with a TESLA BS 540 microscope operating at 80 kV with *λ* = 0.00418 nm. Returning to the same position of the stage plate was solved by the electric steering and stage control unit allowing the precise identification of cells and nanoparticles. The conversion and computer direction of the TESLA BS microscope to iTEM was described earlier [5].

### 4.5. Scanning Electron Microscopy (SEM)

Purified lactomicroselenium particles were fixed for 30 min at 0 °C with 2.5% glutaraldehyde in PBS. After washing with the same buffer nanoparticles were fixed for a further 30 min at 0 °C with 2.5% osmium tetroxide in the same buffer. Nanoparticles were dehydrated stepwise, using increasing concentrations of ethanol in the rage of 50%–100% and were coated with gold for viewing by a Hitachi S 4300 scanning electron microscope (Schaumburg, IL, USA).

### 4.6. Sofware

The server software was sending information gathered by the camera from the microscope through the internet to client computers, while local control of the microscope was also maintained. The server application passed and received text messages with client computers, opening a chat-like channel between the operator and users. Frequently-used command phrases related to the local control of the microscope were available as keystroke shortcuts for convenient use. The local operator granted the right of control to clients. Supervision of less-experienced remote users served to protect the sample and microscope. While controlling the electron microscope remotely, desynchronization between the video stream and control keystrokes could have occurred. The lagging depended on two major parameters: (a) a significant source of shift came from the communication time between the two computers, known as “ping-time”. This could not be controlled, since it was a physical parameter of the network between the computers; and (b) the second parameter was the time of the analog/digital conversion of the video signal via the hardware. By optimizing the control system, this frame-lapse could be reduced to 100 ms. To improve the overall performance of the system, several further changes have been made. Converting the 720 × 576 pixel frames to single channel-8-bit grayscale, reduced the necessary bandwidth to 40%. We have also incorporated a so-called “quick-mode” by using image compression and reduced image enhancement with remote users cruising around the specimen. When the region of interest was selected, the software switched back to the uncompressed, enhanced mode of operation.
